# SNP application improves drought tolerance in soybean

**DOI:** 10.1038/s41598-023-38088-8

**Published:** 2023-07-05

**Authors:** Qi Zhou, Yumei Tian, Xiaomei Li, Zihao Wu, Xiyue Wang, Shoukun Dong

**Affiliations:** 1 Faculty of Agriculture, Northeast Agricultural University, Xiangfang District, Harbin, 150030 China; 2 Agriculture and Food Science and Technology Branch, Heilongjiang Agricultural Engineering Vocational College, Nangang District, Harbin, 150025 China

**Keywords:** Plant physiology, Plant stress responses

## Abstract

As an important bioactive molecule, nitric oxide (NO) can effectively alleviate the effects of drought stress on crops. However, it is still unclear whether it can increase the stress resistance of soybean. Therefore, in this study, our objective was to explore the effect of exogenous NO application on the physiological characteristics of soybean seedlings under drought stress. As test material, two soybean varieties, HN65 and HN44, were used, while sodium nitroprusside (SNP) of 100 μmol L^−1^, 200 μmol L^−1^, 500 μmol L^−1^, 1000 μmol L^−1^ served as an exogenous NO donor, and PEG-6000 as an osmotic regulator to simulate drought stress. The effects of irrigation with different SNP concentrations for different days on the physiological characteristics of the soybean seedlings under drought conditions were then investigated. The results obtained showed that the activities of antioxidant enzymes, osmotic regulator contents, as well as the abscisic acid and salicylic acid contents of the plant leaves increased with increasing SNP concentration and treatment time. However, we observed that excessively high SNP concentrations decreased the activities of key nitrogen metabolism enzymes significantly. This study provides a theoretical basis for determining a suitable exogenous NO concentration and application duration. It also highlights strategies for exploring the mechanism by which exogenous NO regulates crop drought resistance.

## Introduction

Soybean (*Glycine max* L. Merr.), an annual herb originating from the Yellow River Basin of China, has been cultivated for more than 3000 years^1,2^. This plant, which is rich in nutrients, is an important economic crop that also serves as an oil source and is used in the production of high-quality feed for livestock^3^. Soybean is the fourth most cultivated food crop in the world, and in 2019, its production, globally exceeded 300 million tons^4^. Even though it shows considerable robustness and can be cultivated around the world, its growth and development are highly vulnerable to various abiotic and biotic stresses^3^, and specifically, among abiotic stresses, it is most affected by drought, which causes over 40% yield loss^5^.

Drought stress significantly reduces soil water potential, inhibits material transport, and induces the production of large amounts of reactive oxygen species (ROS)^6^. Reportedly, superabundant ROS production can cause oxidative damage to plants, lead to plant metabolic disorders, affect crop growth and development, and in severe conditions, even cause plant death^7^. To reduce ROS-induced oxidative damage, plants maintain their normal metabolism by upregulating the activity of antioxidant enzymes and increasing the content of osmotic regulators^8^. The increase in the contents of intracellular osmotic adjustment substances, e.g., soluble sugars (SS), soluble proteins (SP), and proline (Pro) can effectively reduce water loss. Pro also has several other functions. For example, it protects the cell membrane and scavenges superoxide anion free radicals^9^. Antioxidant enzymes and osmotic adjustment substances can effectively remove excessive ROS in plant cells and reduce or eliminate oxidative damage^7^.

In addition to increasing the content of osmotic adjustment substances as well as antioxidant enzyme activity, plants also have other mechanisms by which the resist adversity, with nitrogen metabolism and endogenous hormones playing important roles in this regard. Nitrogen metabolism is not only closely related to plant growth and yield, but is also a vital player in plant tolerance to abiotic stresses^10^. Additionally, it has been observed that endogenous hormones play a vital regulatory role in plant growth. The content and proportion of endogenous hormones in plants can regulate plant metabolic processes and minimize the adverse effects of drought stress^11^. For example, an increase in abscisic acid (ABA) content can regulate stomatal closure, reduce water loss, decrease net photosynthetic rate, and alleviate drought-induced damage^12^. It has also been reported that salicylic acid (SA) accumulation can reduce oxidative damage and increase the drought tolerance of plants under drought conditions^13^.

It was also recently demonstrated that under abiotic stress conditions such as drought, NO can directly scavenge ROS as an antioxidant^14^ or indirectly scavenge them by enhancing antioxidant enzyme activity^15^. And, NO can also improve plant stress resistance^16^. However, in one previous study, it was observed that high concentrations of NO can damage the nitrogen metabolism system of plants and significantly inhibit the activity of key nitrogen metabolism enzymes^17^. It is also still unclear whether exogenous NO can increase the stress resistance of soybean and affect endogenous hormones and nitrogen metabolism^18^.

Therefore, in this study, our objective was to explore the effects of exogenous NO on the physiological properties and key endogenous hormones in soybean under drought conditions. To this end, two varieties of soybean, Hei Nong65 (HN65) and Hei Nong44 (HN44), with different abilities to resist stresses, such as droughts were selected as test materials. Further, PEG-6000 was used as an osmotic substance regulator to simulate drought stress, while sodium nitroprusside (SNP) was used as the exogenous NO donor. Thus, we hope to provide a theoretical basis for selecting a suitable concentration and method for exogenous NO application during the later stage of soybean development. We also hope that our findings will facilitate future studies to explore the mechanism by which exogenous NO regulates crop drought resistance.

## Results

### Effects of different SNP concentrations on NO concentration in soybean leaves under drought stress

The NO content of HN44 leaves increased significantly under drought stress. Our results also indicated that the NO concentration in soybean leaves increased with the extended of treatment time at 100, 200, 500, 1000 μmol L^−1^ SNP concentration. The NO concentration in soybean leaves increased with the increase of SNP concentration at 1, 3, 5 and 7 days after treatment. The NO contents of HN44 leaves following the D + SNP1000 treatment on days 1, 3, 5, 7 d were 137.11, 106.62, 51.79, and 71.91% higher than those corresponding to the drought-only treatment, respectively, with the differences being statistically significant (Table 1).

**Table 1:** Changes of nitric oxide concentration following different treatments.

NO Content (nmol g^−1^)	HN44				HN65			
Treatment	Time (d)							
	1	3	5	7	1	3	5	7
CK	177.78^e^	235.33^e^	243.44^e^	224.89^e^	124.22^e^	271.22^c^	330.89^d^	359.56^f^
D	241.00^d^	286.67^cd^	369.87^d^	443.70^d^	142.11^e^	261.67^c^	370.26^d^	407.60^e^
D + SNP100	339.56^c^	300.89^cd^	427.78^c^	476.16^cd^	220.67^d^	240.00^c^	461.66^c^	437.78^d^
D + SNP200	373.67b^c^	310.11^c^	437.44^b^	489.50^c^	283.00^c^	373.00^b^	540.33^b^	541.89^c^
D + SNP500	396.89^b^	409.44^b^	468.15^bc^	629.89^b^	343.00^b^	550.89^a^	635.11^a^	736.33^b^
D + SNP1000	571.44^a^	592.33^a^	561.44^a^	762.78^a^	456.44^a^	537.11^a^	684.44^a^	907.33^a^

The superscript letters represent values showing a significant difference relative to the indigenous level at a 5% significance level. CK, control treatment; D, drought treatment; HN44, Hei Nong44; HN65, Hei Nong65; D + SNP100, D + SNP200, D + SNP500, and D + SNP1000, D + SNP treatment at 100, 200, 500, and 1000 μmol L^−1^, respectively.

Further, there was no significant difference between the CK- and D-treated HN65 leaves with respect to NO content. Similar to HN44, the NO concentration in HN65 leaves increased with an increase in SNP concentration and treatment time. Specifically, the NO contents of HN65 leaves following the D + SNP1000 treatment were 221.19, 105.26, 71.35, and 122.60% higher than those corresponding to the drought-only treatment on days 1, 3, 5, and 7, respectively, and the differences were statistically significant (Table 1). Our results showed that variety, time, treatment, variety * time, variety * treatment, variety * time * treatment all caused significant differences in NO content (Table 2).

**Table 2:** Multi-factor interaction analysis.

Significance	Variety	Time	Treatment	Variety * Time	Variety * Treatment	Time * treatment	Variety * Time * Treatment
NO content	0.040	0.000	0.000	0.000	0.000	0.000	0.000
SOD Activity	0.000	0.000	0.000	0.014	0.249	0.003	0.825
POD Activity	0.000	0.000	0.000	0.120	0.000	0.000	0.001
APX Activity	0.000	0.000	0.000	0.000	0.000	0.000	0.000
Pro Content	0.000	0.000	0.000	0.000	0.000	0.000	0.000
SS Content	0.000	0.000	0.000	0.000	0.000	0.000	0.000
SP Content	0.000	0.000	0.000	0.000	0.000	0.000	0.000
NR Activity	0.000	0.000	0.000	0.002	0.000	0.000	0.000
NiR Activity	0.000	0.000	0.000	0.000	0.000	0.000	0.000
ABA Content	0.000		0.000		0.000		
SA Content	0.000		0.000		0.000		

Note: The table shows the influence of different factors and the influence of different factors on physiological indexes. Significance less than 0.05 indicates that a certain factor or the interaction between different factors has a significant impact on a certain indicator.

### Effects of different SNP concentrations on the antioxidant capacity in soybean leaves under drought stress

**Figure 1:**
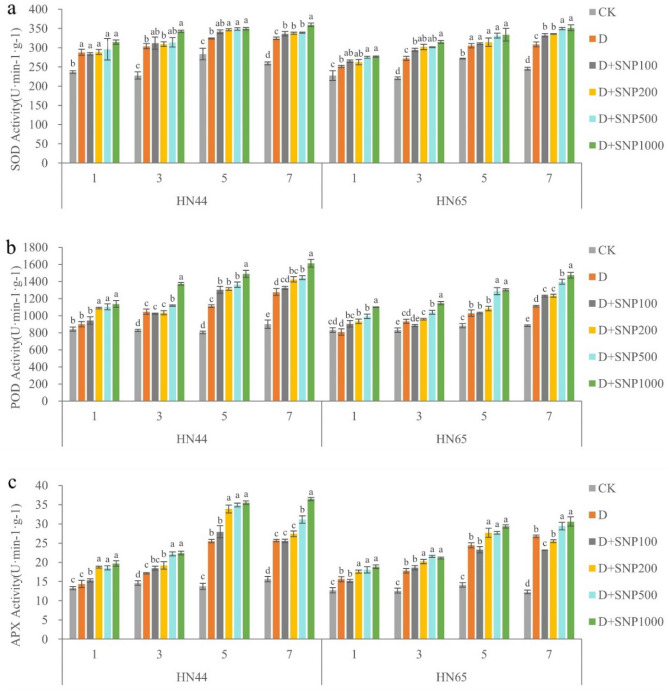
The different letters represent values showing a significant difference relative to the indigenous level at a 5% significance level. Changes in superoxide dismutase (SOD), peroxidase (POD), and ascorbate peroxidase (APX) activities under different sodium nitroprusside (SNP) treatments. (CK, control treatment; D, drought treatment; HN44, Hei Nong44; HN65, Hei Nong65; D + SNP100, D + SNP200, D + SNP500, and D + SNP1000, D + SNP treatment at100, 200, 500, and 1000 μmol L^−1^, respectively).

As shown in Fig. 1, drought stress resulted in a significant increase in SOD, POD, and APX activities in HN44 leaves. Further, under drought conditions, exogenous NO further improved the activities of SOD, POD, and AXP, and with an increase in exogenous NO content, enzyme activity gradually increased. Specifically, on days 1, 3, 5, and 7, all the peak SOD, POD, and APX activity levels observed resulted from the SNP1000 treatment. In HN44 leaves on days 1, 3, 5, and 7 and under the D + SNP1000 treatment, SOD activities were 9.35, 12.73, 7.79, and 10.81% higher than those observed under the drought-only treatment, POD activities were 26.22, 31.31, 34.20, and 26.46% higher than those observed following the drought-only treatment, respectively, and APX activities were 37.05, 30.40, 39.44, and 42.14% higher than those corresponding to the drought-only treatment, respectively.

Similarly, drought stress significantly increased SOD, POD, APX activities in HN65 leaves, and exogenous NO application further improved these SOD, POD, and AXP activities under drought conditions. As SNP concentration increased, antioxidant enzyme activities also gradually increased. Specifically, considering all the SNP treatments durations, days 1, 3, 5, and 7, maximum SOD, POD, and APX activities were always observed under the D + SNP1000 treatment. We also observed that for HN65 on days 1, 3, 5, and 7 under the D + SNP1000 treatment, the SOD activities were 10.20, 15.67, 9.41, and 13.90% higher than those observed under the drought-only treatment, POD activities were 35.85, 22.92, 26.59, and 32.46% higher than those observed under the drought-only treatment, respectively, and APX activities were 20.83, 18.77, 20.10, and 14.34% higher than those observed under the drought-only treatment, respectively.

In summary, SOD, POD, and APX activities increased with increasing SNP concentration and application time, indicating that exogenous NO could enhance the drought resistance of HN65 and HN44 by enhancing antioxidant enzyme activity. It was also evident that antioxidant enzyme activities in the leaves of the soybean varieties were significantly different under the same conditions. After treatment with SNP1000 for 7 days, the POD and CAT activities in HN44 leaves were significantly higher than those in HN65 leaves, respectively. However, the SOD activity in HN44 not significantly different from that in HN65. Our results also found that the three single factors of variety, treatment and time had significant effects on SOD, POD and APX activities. Only the interaction of variety * time and treatment * time had a significant effect on SOD activity. The interaction of variety * treatment, treatment * time and variety * treatment * time had a significant effect on POD activity. Variety * treatment, variety * time, treatment * time, variety * treatment * time had a significant effect on APX activity (Table 2).

### Effects of different SNP concentrations on osmotic regulation capacity in soybean leaves under drought stress

**Figure 2:**
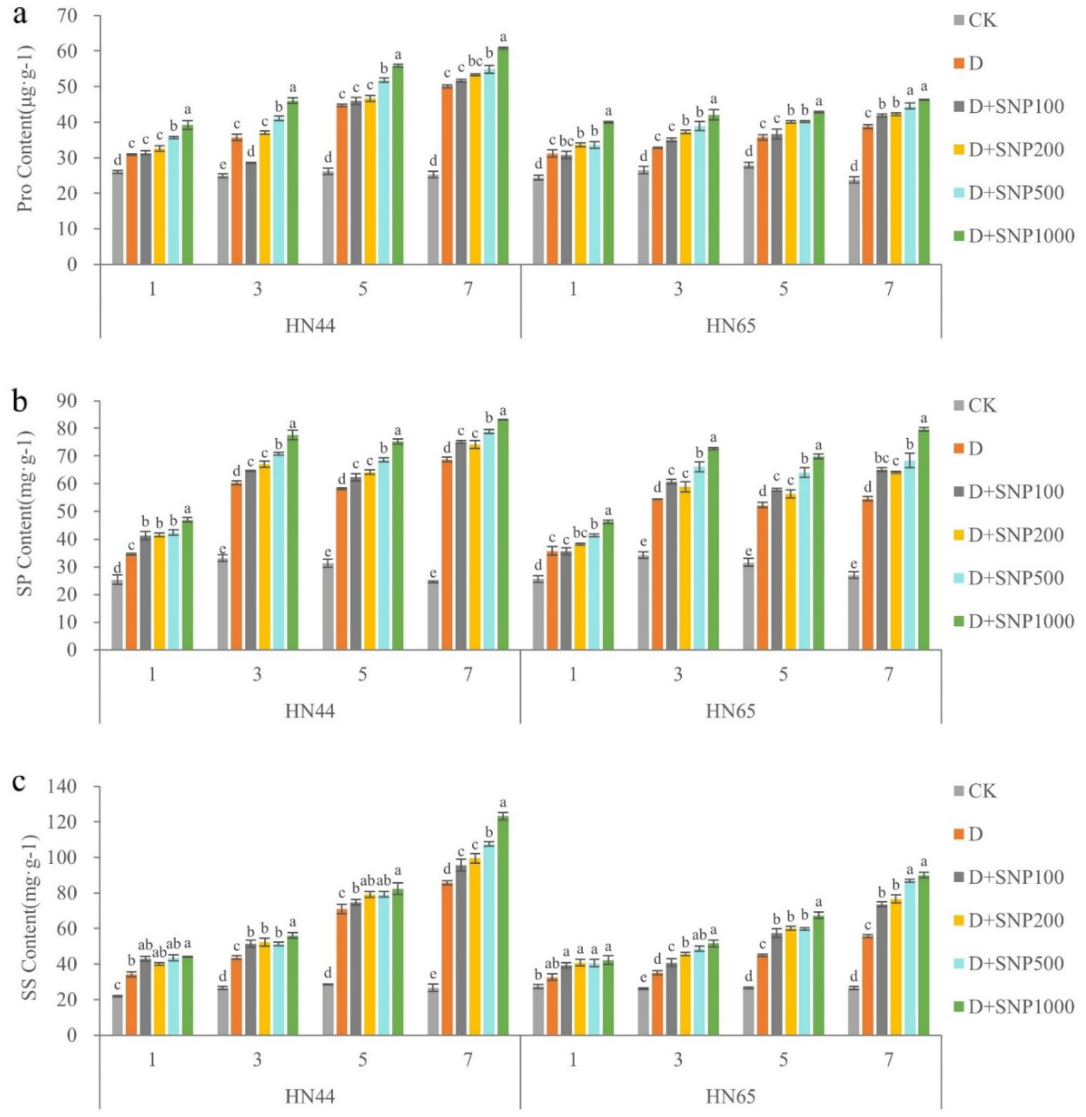
The different letters represent values showing a significant difference relative to the indigenous level at a 5% significance level. Changes in proline (Pro), soluble protein (SP), and soluble sugar (SS) concentrations under different sodium nitroprusside (SNP) treatments. (CK, control treatment; D, drought treatment; HN44, Hei Nong44; HN65, Hei Nong65; D + SNP100, D + SNP200, D + SNP500, and D + SNP1000, D + SNP treatment at100, 200, 500, and 1000 μmol L^−1^, respectively).

Pro, SP, and SS concentrations in HN44 leaves increased with increasing SNP concentration during the whole drought stress period, reaching a maximum in the SNP1000-treated plants (Fig. 2). Further, on days 1, 3, 5, and 7, the maximum Pro, SP, and SS concentrations were always observed under the SNP1000 treatment. Further, when HN44 plants were subjected to the D + SNP1000 treatment for 1, 3, 5, and 7 days, the Pro concentrations obtained were 26.86, 28.75, 24.84, and 21.48% higher than those observed under the drought-only treatments, respectively. Further, the SP concentrations were 35.86, 28.71, 29.07, and 20.83% higher than those observed under the drought-only treatments, respectively, and the SS concentrations were 29.12, 28.36, 16.14, and 43.86% higher than those observed under the drought-only treatment, respectively.

In HN65 leaves, Pro, SP, and SS concentrations also increased with increasing SNP concentration during the whole drought stress period (Fig. 2). Specifically, for HN65 under the D + SNP1000 treatment, on days 1, 3, 5, and 7, the observed Pro concentrations were 28.35, 28.32, 19.64, and 19.40% higher than those corresponding to the drought-only treatment, respectively. Further, the observed SP concentrations were 29.71, 33.42, 33.69, and 45.93% higher than those observed under the drought-only treatment, respectively and the SS concentrations were 29.59, 47.45, 50.19, and 62.01% higher than those observed under the drought-only treatment, respectively.

In summary, different SNP concentrations resulted in varying Pro, SP, and SS concentrations, indicating that exogenous NO application can enhance the drought resistance of HN44 and HN65 soybean varieties by increasing the osmotic regulation capacity of the plants. It was also evident that under similar treatment conditions, HN44 always showed a higher concentration of osmotic regulation substances than HN65. After 7 days of drought treatment without exogenous NO, the Pro, SP, and SS concentrations in HN44 were 29.13, 26.03, and 54.07% higher than those observed for HN65, respectively. We also observed that an SP concentration of 83.14 mg·g^−1^ for HN44 on day 7 following the D + SNP1000 treatment. This was not significantly different from that observed for HN65. On day 7, the Pro and SS concentrations observed for HN44 following the D + SNP1000 treatment were significantly higher than those observed for HN65. We also observed that variety, treatment, time, variety * treatment, variety * time, treatment * time, variety * treatment * time have a significant impact on Pro, SS, SP (Table 2).

### Effects of different SNP concentrations on the activities of key nitrogen metabolism enzymes in soybean under drought stress

**Figure 3:**
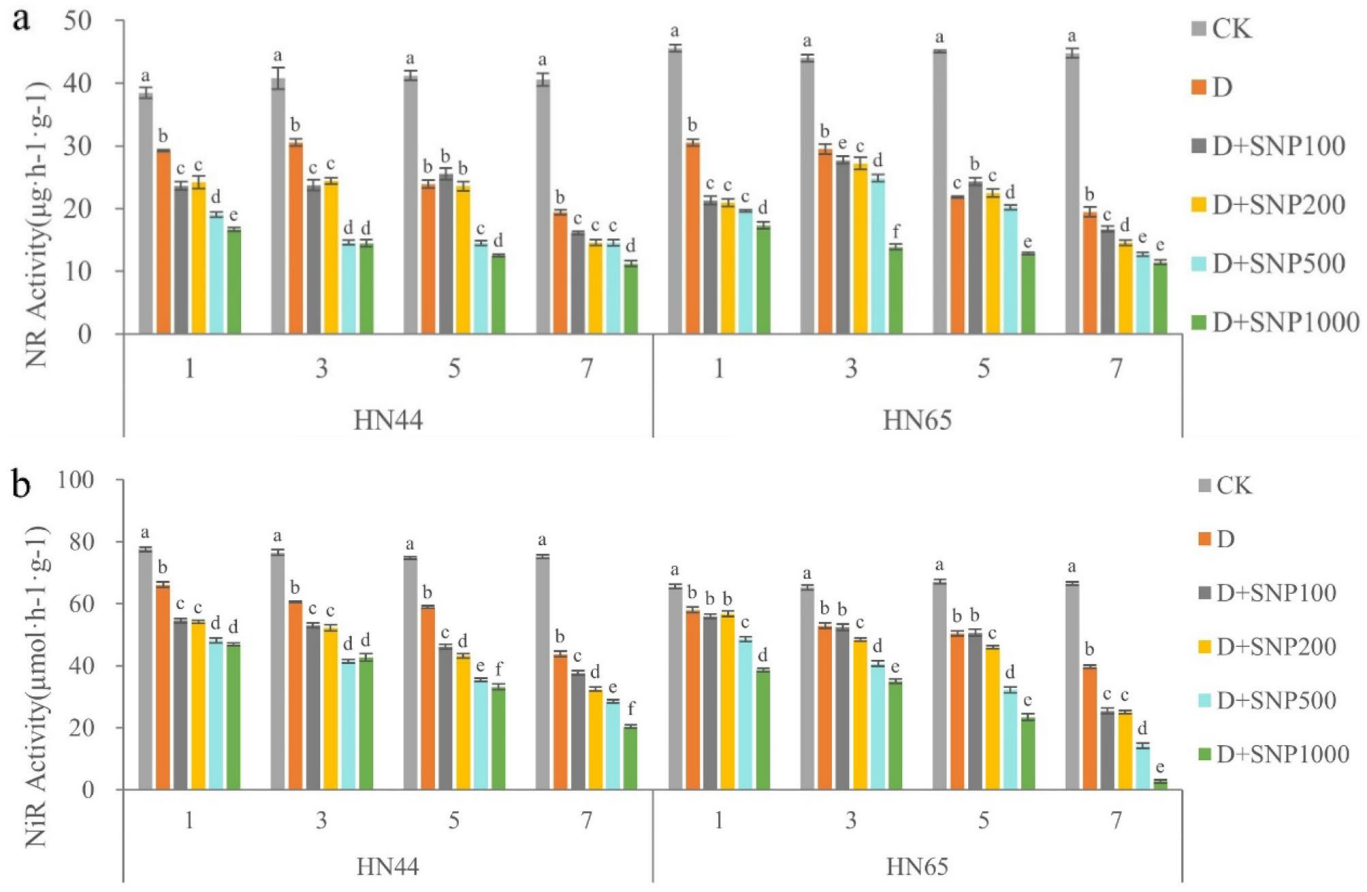
The different letters represent values showing a significant difference relative to the indigenous level at a 5% significance level. Changes of nitrate reductase (NR) and nitrite reductase (NiR) activities under different sodium nitroprusside (SNP) treatments. (CK, control treatment; D, drought treatment; HN44, Hei Nong44; HN65, Hei Nong65; D + SNP100, D + SNP200, D + SNP500, and D + SNP1000, D + SNP treatment at100, 200, 500, and 1000 μmol L^−1^, respectively).

The changes of NR and NiR activities in HN44 leaves under different SNP concentrations are shown in Fig. 3. From this figure, it is evident that an increase in SNP concentration and treatment duration resulted in decreased NR and NiR activities under drought conditions. Under the D + SNP1000 treatment for 7 days, NR and NiR showed the lowest activities. Further, compared with SNP concentration, drought time had a greater effect on NiR activity. We also observed significant differences between each SNP concentration on days 5 and 7.

Under the drought condition, NR and NiR activities in HN65 leaves under the different SNP concentrations (Fig. 3) decreased gradually with increasing SNP concentration. Notably, the lowest NR activity was observed under the D + SNP1000 treatment. Under this treatment on days 1, 3, 5, and 7, NR activities were 43.26, 52.93, 41.02, and 41.13% lower than those observed under the drought-only condition, respectively. With the extension of drought time, NiR activity also decreased gradually, and with increasing SNP concentration, NiR activity decreased further. Specifically, the activity of NiR was 2.71 μmol h^−1^ g^−1^ under the D + SNP1000 treatment on day 7, 95.90% lower than that observed under the drought-only treatment on day 1.

In summary, SNP irrigation of high concentrations under drought conditions significantly decreased NR and NiR activities. Additionally, our results also indicated different NR activities for HN44 and HN65 before day 3; however, the differences decreased or even disappeared by days 5 and 7 of treatment. Further, the NiR activity observed for HN65 was significantly lower than that observed for HN44 except under the D + SNP1000 treatment. We also observed lower NiR activity for HN44 than HN65 within the first three days of treatment. However, on day 5, there was no significant difference between them in this regard. The NiR activity of HN65 was significantly lower than that of HN44 on day 7 of treatment. We also observed that variety, treatment, time, variety * treatment, variety * time, treatment * time, variety * treatment * time have a significant impact on NR, NiR (Table 2).

### Effects of different SNP concentrations on endogenous hormone contents of soybeans under drought stress

**Figure 4:**
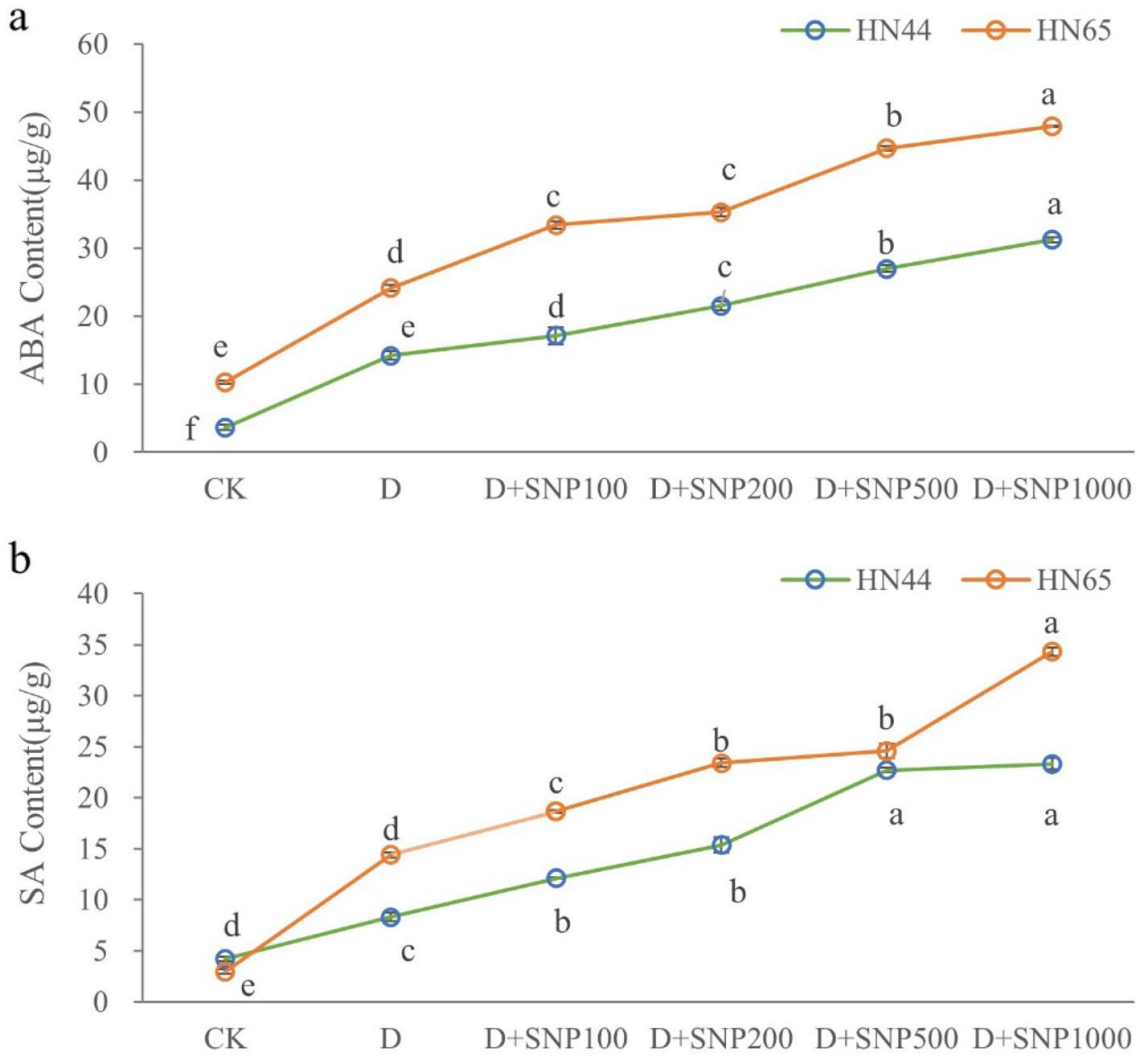
The different letters represent values showing a significant difference relative to the indigenous level at a 5% significance level. Changes in abscisic acid (ABA) and salicylic acid (SA) contents under different SNP treatments by day 7. (CK, control treatment; D, drought treatment; HN44, Hei Nong44; HN65, Hei Nong65; D + SNP100, D + SNP200, D + SNP500, and D + SNP1000, D + SNP treatment at100, 200, 500, and 1000 μmol L^−1^, respectively).

Based on the measured physiological indicators, it was evident that the differences between the different treatments were the most significant on day 7. Thus, we measured ABA and SA contents for HN65 and HN44 under the different treatments on day 7. The results thus obtained are shown in Fig. 4. From this figure, it is evident that the ABA content of HN44 leaves increased significantly under drought stress, and further increased with increasing SNP irrigation. Specifically, ABA content increased with increasing SNP concentration, and the maximum ABA content of HN44 leaves was observed under the D + SNP1000 treatment. HN65 and HN44 showed similar changes. However, the difference between the ABA contents observed for HN65 and HN44 was that for HN65, there was no significant difference between the D + SNP100 and 200 treatments, while these two treatments resulted in different ABA contents for HN44. Our results also indicated that under all the different treatments, HN65 always showed higher ABA contents than HN44.

Our results also indicated that drought significantly increased SA concentration in HN44 and HN65 leaves. Except when the SNP concentrations were 200 and 500 μmol L^−1^, no significant differences in SA content of HN65. And except when the SNP concentrations were 100 and 200 μmol L^−1^, no significant differences in SA content of HN44. Under other treatment condition, with increasing SNP concentration, SA contents increased, and under the D + SNP1000 treatment, we observed the maximum SA content. In general, the SA content of HN65 leaves was lower than that of HN44 leaves under the CK treatment; however, the SA content of HN44 leaves was significantly lower than that of HN65 leaves after the drought and SNP treatments, indicating that HN65 was more sensitive to drought than HN44. We also observed that variety, treatment, variety * treatment have a significant impact on SA, ABA (Table 2).

### Pearson correlation analysis and principal component analysis (PCA)

**Figure 5:**
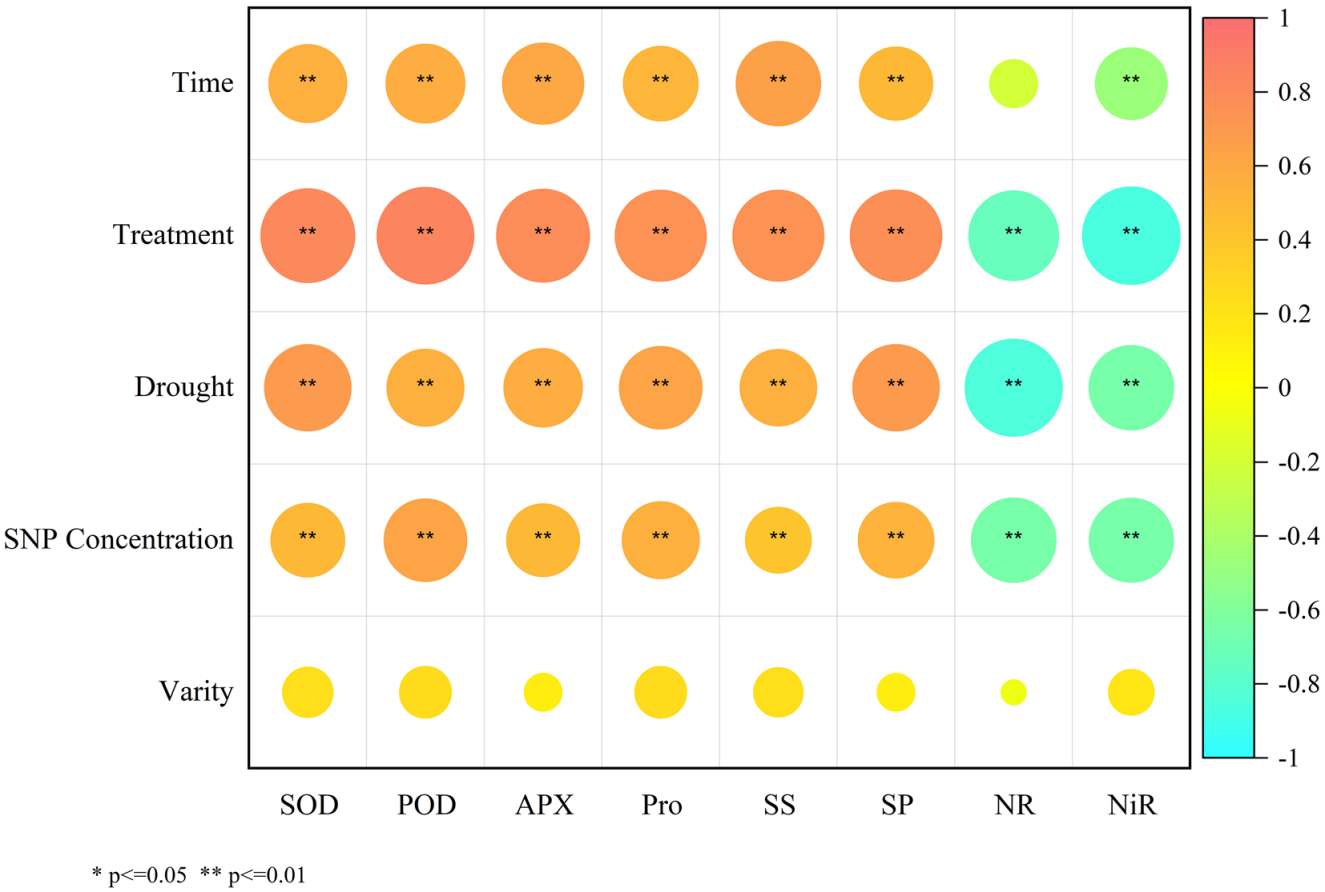
Pearson correlation analysis. Note: The correlations between eight physiological indexes and treatment time, NO content, drought or not, SNP concentration and variety are shown in the figure. The values in the figure are *P*-values. The *P*-value closer to 1 means stronger positive correlation, closer to 0 means no correlation, closer to -1 means stronger negative correlation.

We analyzed the correlation between the eight physiological indexes and different treatment conditions (as shown in Fig. 5), and found that the correlation between physiological indexes and varieties was the smallest, and the correlation with NO content was the largest. The results showed that except NR activity, the other seven physiological indices were significantly correlated with SNP concentration, drought condition, NO content and treatment time. NR activity was also significantly correlated with NO content, drought and SNP concentration. The correlation between all physiological indexes and varieties did not reach a significant level. It shows that the application of exogenous NO under drought conditions will have a very significant effect on the physiological status of soybean, and this effect is not significantly different between varieties.

**Figure 6:**
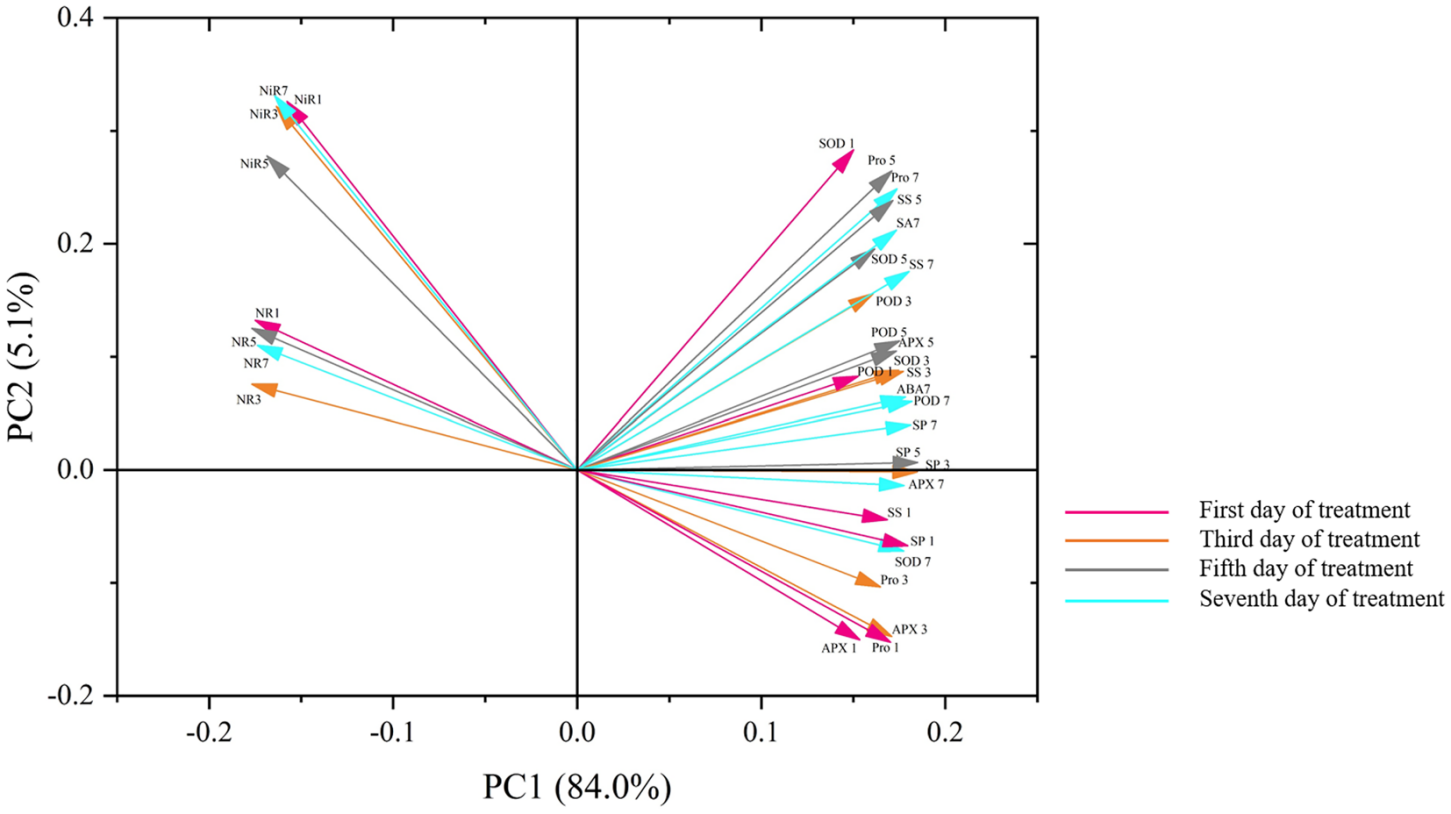
Principal component analysis results. (PC1, principal component 1; PC2, principal component 2).

To better visualize the results obtained in this study, we performed PCA. The results thus obtained are shown in Fig. 6, from which it is evident that PC1 accounted for 84.0% of the observed results, while PC2 accounted for 5.1% of the observed results. The overall separation index was greater than 80%, indicating that the results were good. Further, PC1 was primarily related to the different indicators corresponding to the different exogenous SNP concentrations, while PC2 was primarily related to the varieties, accounting for only 5.1% of the total variance. This indicated that under different exogenous SNP concentrations, NR, NiR, SOD, POD, and APX activities and Pro, SP, SS, ABA, and SA contents had little relationship with the soybean varieties. It can also be seen from the correlation analysis that the correlation between each physiological index and the variety is small, indicating that the difference between the two varieties under different treatments is small. These results also indicated that under exogenous NO application, the drought resistance of the more drought-sensitive variety, HN65, was effectively enhanced, such that it was comparable to that of the more drought-resistant variety, HN44.

## Discussion

Drought stress, which often inhibits plant growth rate, is accompanied by visible damage, such as leaf yellowing^19^. In addition to such visible damage, it can also induce the excessive accumulation of toxic ROS, leading to oxidative stress, which destroys cellular structures, damages biological macromolecules, such as lipids, proteins, and DNA, and ultimately leads to cell death^20^. Studies have shown that under abiotic stresses, including drought, an increase in NO-mediated osmotic regulation and antioxidant capacity is essential for scavenging excessive ROS and improving plant drought resistance^18^. Moreover, NO can also enhance the absorption of mineral nutrients by plants, regulate the content of various key plant hormones, and improve photosynthetic efficiency and biomass accumulation, such that plants can maintain an optimal growth state under stress conditions^15,21^. In this study, we used SNP as an exogenous NO donor, and observed that with increasing SNP concentration and treatment time, leaf NO content increased. We also investigated the differential response of soybean to different SNP concentrations under water shortage conditions, and examined the mitigation effect of SNP on soybean drought stress.

Previous studies have shown that the accumulation of antioxidant enzymes in plants under drought stress conditions helps to maintain ROS metabolic balance, effectively stabilize cell structure, and protect intracellular biological macromolecules from ROS toxicity^22^. In this study, we observed that drought stress induced a significant increase in antioxidant enzyme level, and exogenous NO application during drought stress further improved the antioxidant enzyme activity. Tian and Lei^23^ and Yan^24^ arrived at a similar conclusion, suggesting that exogenous NO could improve the dehydration tolerance of plants by increasing the activities of antioxidant enzymes under drought conditions, and effectively reduce the contents of H_2_O_2_ and superoxide anion radicals in plant cells, and reduce oxidative damage. However, some studies have shown that NO has a limited ability in regulating plant antioxidant activity and photosynthesis under abiotic stress. For example, it was previously reported that during the reproductive growth stage of plants, an increase in SNP concentration results in antioxidant enzyme activity eventually tending to a critical value^25,26^. In this study, we arrived at a similar finding. However, it is also believed that under short-term (e.g., 7 days) drought stress, an increase in exogenous NO content and the prolongation of treatment time results in a gradual increase in the levels of antioxidant enzymes, such as SOD, POD, and APX, which eventually tend to be stable or may increase first and thereafter decrease. Further, the levels of these antioxidant enzymes are closely related to plant growth habits and variety types^27^.

Reportedly, the accumulation of osmotic regulators, such as Pro, SS, and SP can help maintain a high-water potential as well as osmotic pressure in cells. This could effectively lead to the prevention of cell dehydration and the improvement of cell membrane stability under drought conditions^6^. Further, it is generally believed that the content of osmotic adjustment substances under drought conditions is significantly associated with the drought tolerance of plants. Further, drought-tolerant crops have higher osmotic adjustment abilities^28^. Xu et al.^29^ considered that applying exogenous regulators to plants under stress conditions is an important strategy for improving plant stress resistance. For example, applying exogenous NO to plants at different growth stages can significantly lead to an increase in the concentration of osmotic regulators in plant cells, prevent cell water loss, and significantly improve plant drought tolerance. Based on our results in this study, we arrived at a similar conclusion that the content of osmotic adjustment substances in plants increases significantly after exogenous NO application. We also observed that their levels were lower in a drought-sensitive soybean variety, HN65, than in the drought-resistant, variety HN44, under different treatment conditions. Additionally, in this study, the content of osmotic adjustment substances increased with an increase in SNP concentration. Xiu et al.^26^ also reached a similar conclusion in a study involving peanuts, and considered that to a certain extent, the application of different concentrations of SNP under abiotic stress could protect plant membrane lipids and proteins against oxidative damage by increasing the content of osmotic regulators.

NR and NiR, as key enzymes in plant nitrogen metabolism, can reflect the overall level of nitrogen assimilation and protein synthesis in plants. They also play a vital role in plant growth and development^6,30^. In this study, our results showed that the activities of nitrogen metabolism enzymes, such as NR, were significantly reduced under drought stress. Liang et al.^6^ reported that this observation may be due to an increase in hydrolase activity and a decrease in substrate content, resulting in the inhibition of protein synthesis. Majeed et al.^18^ suggested that exogenous growth regulators, e.g., NO, can induce significant increases in NR and NiR activities in maize under drought stress, and that NR and NiR activities increase with increasing exogenous NO content. However, in this study, under 500 and 1000 μmol·L^−1^ SNP treatments, NR and NiR activities decreased significantly, and possibly caused some damage to the nitrogen metabolism system. Thus, we speculated that this may be related to the different experimental materials used as well as differences in treatment duration. In their studies involving wheat and Chlamydomonas reinhardtii, Eliana et al.^17^ and Sanz-Luque et al.^31^ reported that NR and NiR activities are negatively regulated by SNP-released NO. They also observed that this negative regulation was more pronounced at higher NO concentrations. This is consistent with our experimental results. Additionally, Eliana et al.^17^ reported that this observation possibly resulted from a decrease in the number of NOA1/RIF1 proteins or the excessive accumulation of NO^3-^ and NO, leading to the labeling of enzyme molecules and resulting in a decrease in activity. Notwithstanding, this precise negative regulation and action pathway is still inconclusive.

ABA and SA are involved in plant response to abiotic stress. Specifically, SA helps to reduce oxidative damage in plants and ABA can reduce the stomatal conductance of plants to prevent cell water loss. The accumulation of both substances under drought conditions can improve plant drought tolerance^4^. Our results showed that exogenous NO application could effectively increase ABA and SA contents under drought conditions. We also observed that ABA and SA contents in HN44 were significantly higher than those in HN65. Jing et al.^25^ reported that exogenous NO can induce the accumulation of endogenous hormones, such as ABA, during the vegetative growth stage of plants to improve drought tolerance; this is consistent with our research results. However, Jing et al.^25^ further reported that there is a very complex metabolic contradiction between the metabolism of endogenous hormones, such as NO and ABA, and the metabolic processes are very different at different growth stages and under different stress conditions. These differences remain to be further investigated.

In summary, our findings suggested that SNP application under drought conditions can help increase the drought resistance of soybeans; however, at high concentrations, SNP can also cause serious toxicity to the nitrogen metabolism system of soybeans. Further, based on our findings, we identified an SNP concentration of 200 μmol L^−1^ for a treatment duration of 5 days as most suitable to compensate for the damage on soybean growth and development caused by drought conditions. When the SNP concentration exceeded 200 μmol L^−1^, possibly, the nitrogen metabolism system was seriously damaged. Therefore, to alleviate drought stress-induced damage to crops and improve the economic benefits associated with the crop cultivation, it is necessary to select an appropriate SNP application duration and concentration taking into consideration actual production conditions and differences between species and varieties. Additionally, to clarify how exogenous NO regulates drought resistance in soybean, the activity of nitrogen metabolism enzymes and the complex interaction between NO and endogenous hormones, in future studies, we will analyze molecular as well as metabolic regulation in this regard to the end of providing a theoretical basis for soybean cultivation and breeding.

## Materials and Methods

### Testing material

In this study, which was conducted at the Northeast Agricultural University of China (N45° 44′ 43.87, E126° 43′ 50.42), HN65 and HN44, bred by the Soybean Research Institution of Heilongjiang Academy of Agricultural Sciences, China, were selected as test materials. A previous study showed that HN65 shows weaker drought resistance than HN44^32^. Our research team obtained permissions to cultivated soybean plants from the Soybean Research Institution of Heilongjiang Academy of Agricultural Sciences, China to this study. The plant materials are not wild plants or endangered species. Experimental research and field studies on plants including the collection of plant material, complied with relevant institutional, national, and international guidelines and legislation.

### Experimental design

The experiments in this study were conducted in a ventilated greenhouse. In brief, clean fine sand was place in 40-cm high and 24-cm diameter flowerpots. Thereafter, each pot is planted with six seeds of the same size, and 500 mL of water was added to each pot daily. When the opposite euphylla were fully expanded, three seedlings showing the same growth vigor were retained in each pot. After thinning, 500 mL of Hoagland nutrient solution^33^ was irrigated into each pot daily until all the seedling sprouts grew two leaflets.

Six treatments were applied in this study. The control treatment (CK) involved irrigation with 500 mL of nutrient solution daily, while the drought treatment (D) involved irrigation with 500 mL of 15% PEG-6000 nutrient solution daily (The water potential is about -0.33 MPa). The remaining treatment groups involved the addition of 100, 200, 500, and 1000 μmol SNP to the 15% PEG-6000 nutrient solution per liter; thus, the SNP concentrations corresponding to these four treatments were 100, 200, 500, and 1000 μmol L^−1^, respectively. Further, these four treatment groups were named: D + SNP100, D + SNP200, D + SNP500, and D + SNP1000, respectively. The watering time was between 06:00 a.m. and 07:00 a.m. every day. Samples were harvested on days 1, 3, 5, and 7 after treatment commencement for analysis. The different treatments were performed in triplicates. The samples were the second leaves at the top of the plants. Additionally, the sampling time was between 10:00 and 11:00 a.m., and after sampling, the samples were stored in an ultra-low-temperature refrigerator at − 80 °C (DW 86L828J; Haier, Qingdao, China).

Thereafter, the contents of osmotic adjustment substances, nitrogen metabolism enzyme activity, and antioxidant enzyme activity were determined. Further, endogenous hormone contents were determined after sample collection on day 7. Triplicate measurements were performed for each indicator.

### Determination of physiological and biochemical indexes

To determine the NO contents of the leaves, 0.10 g of fresh leave tissue sample was placed in a centrifuge tube. This was followed by the addition of 1.00 mL of deionized water. This was followed by boiling in a water bath for 30 min, followed by cooling and boiling again in the water bath for 30 min. Finally, NO content was determined via the Greiss reagent method^34^.

In a cold mortar, 0.10 g of the frozen sample was placed followed by the addition of 1.00 mL of the extraction medium (when measuring peroxidase activity, a small amount of calcium carbonate was added) followed by grinding into a homogenate at 4 °C. Next, centrifugation was performed at 10,000 rpm/min for 15 min, after which the supernatant, which was the crude enzyme extract, was collected, and SOD, POD, and APX activities were determined using the nitrogen blue tetrazolium method, guaiacol method, and ultraviolet spectrophotometry (Junfeng et al., 2006). The extraction medium for SOD was 50 mmol/L phosphate buffer (pH 7.8) containing 1% polyvinylpyrrolidone. For POD, the extraction medium was distilled water, and for APX, it was 50 mmol/L phosphate buffer (pH 7.0) containing 0.10 mmol/L EDTA-Na_2_.

In brief, 0.10 g of the frozen leave tissue sample was placed in a mortar and 1.00 mL of 80% ethanol was added after which the mixture was ground into a homogenate in a water bath at 80 °C for 20 min. Pro content was determined using the acid ninhydrin chromogenic method^35^. Further, to determine SP content, 0.10 g of the frozen sample was placed in a mortar. Thereafter, 1.00 mL of distilled water was added and the mixture was ground into a homogenate followed by centrifugation at 3000 rpm/min for 10 min. Next, the supernatant obtained was directly stained with Coomassie brilliant blue G-250 to determine SP content. For the determination of SS content, boiling was again performed in the water bath for 20 min, after cooling, SS content was determined via anthrone colorimetry^35^.

First, 1.00 g of a fresh plant leave sample was weighed and placed in a small beaker. Thereafter, the sample was pressed to the bottom with a smaller beaker, and 9.0 mL of a 0.1 mol/L solution of KNO_3_ was added (for the control, 1 mL of 0.3 g/mL trichloroacetic acid solution was added prior to the addition of the KNO_3_ solution). Immediately after mixing in a dryer vacuum for 1 min, air was introduced followed by vacuumization, which was repeated several times to eliminate any gas within the tissues until the leaves were completely softened and sank to the bottom of the cup. Thus, the substrate solution entered the tissue. Finally, the reaction mixture was sealed with nitrogen gas and the reaction was allowed to occur in dark at 25 °C for 30 min. Thereafter, 1.00 mL of 0.3 g/mL trichloroacetic acid was added to all the beakers, with the exception of the control beakers, to terminate the reaction. Finally, the activity of NR was determined as previously described Xuekui^36^; Additionally, to determined NiR activity, 0.10 g of the fresh plant leave samples was placed in a cold mortar followed by the addition of 1.00 mL of 0.1 mol/L phosphate buffer (pH 7.5). Next, the mixture was ground into a homogenate, followed by centrifugation at 12,000 rpm/min at 4 °C for 20 min. Finally, NiR activity was then determined according to the Yanli^37^ method.

A 1.00 g frozen sample is ground in an ice bath, 20 mL of 80% pre-cooling methanol is added, sealed, and placed in a 4 °C refrigerator overnight. The extract was filtered and washed twice with 10 mL methanol. After filtration, it was combined with the extract and evaporated at 40 °C until there was no methanol residue. The remaining water phase was transferred to a triangular flask, extracted and decolorized twice with 30 mL pe-troleum ether. The ether phase was discarded. Then 0.01 g PVPP (crosslinked poly-vi-nylpyrrolidone) was added to the ultrasonic for 30 min and filtered. Afterwards, it was extracted with 30 mL ethyl acetate 3 times, combined with the lipid phase, and evapo-rated at 40 °C. The residue was dissolved with methanol (chromatographically pure) and diluted to 2 mL. The test solution was filtered through a 0.45 μm microporous membrane and stored in a refrigerator at 4 °C. Then, according to the method reported by Wang, the contents of abscisic acid (ABA) and salicylic acid (SA), were determined by HPLC^36^.

### Statistical analysis

All statistical analysis were performed using SPSS software (version 21.0: IBM, Armonk, NY, USA), Microsoft Excel 2021 (Microsoft Corp., Redmond, WA, USA) for line chart, and OriginPro 2021 (OriginLab Corp., Northampton, MA, USA) for principal component and pearson correlation analysis.

## Data Availability

The datasets used and/or analyzed during the current study are available from the corresponding author on reasonable request.
